# Application of Plasma Treatment in Preparation of Soybean Oil Factory Sludge Catalyst and Its Application in Selective Catalytic Oxidation (SCO) Denitration

**DOI:** 10.3390/ma11091609

**Published:** 2018-09-04

**Authors:** Lei Zhang, Chao Yang, Lei Zhang, Huibin He, Min Luo, Yang Jia, Yonghui Li

**Affiliations:** 1 School of Geology and Environment, Xi’an University of Science and Technology, Xi’an 710054, China; yyangcchaoo@126.com (C.Y.); lm876098550@126.com (M.L.); qq519322049@126.com (Y.L.); 2 China National Heavy Machinery Research Institute Co., Ltd., Xi’an 710032, China; zlcnhmri@126.com (L.Z.); xzslyh@126.com (Y.L.); 3 Zhejiang Dechuang Environmental Protection Technology Co., Ltd., Xi’an 710000, China; hhbxast@126.com

**Keywords:** selective catalytic oxidation method, sludge, plasma, alkali activation

## Abstract

At present, the most commonly used denitration process is the selective catalytic reduction (SCR) method. However, in the SCR method, the service life of the catalyst is short, and the industrial operation cost is high. The selective catalytic oxidation absorption (SCO) method can be used in a low temperature environment, which greatly reduces energy consumption and cost. The C/N ratio of the sludge produced in the wastewater treatment process of the soybean oil plant used in this paper is 9.64, while the C/N ratio of the sludge produced by an urban sewage treatment plant is 10–20. This study shows that the smaller the C/N ratio, the better the denitration efficiency of the catalyst. Therefore, dried oil sludge is used as a catalyst carrier. The influence of different activation times, and LiOH concentrations, on catalyst activity were investigated in this paper. The denitration performance of catalysts prepared by different activation sequences was compared. The catalyst was characterized by Fourier Transform infrared spectroscopy (FTIR), X-ray diffraction (XRD), X-ray photoelectron spectroscopy (XPS), and scanning electron microscope (SEM). The experimental results showed that: (1) When the concentration of the LiOH solution used for activation is 15%, and the activation time is four hours, the denitration effect of the catalyst is the best; (2) the catalyst prepared by activation before plasma roasting has the best catalytic activity.

## 1. Introduction

At present, the flue gas removal NO_x_ technology that has been studied and applied can be roughly classified into two types: dry flue gas denitration, and wet flue gas denitration. The main methods of dry flue gas denitration are solid adsorption, plasma activation, selective non-catalytic reduction (SNCR), and selective catalytic reduction (SCR) [1,2]. Wet flue gas denitration mainly includes the acid absorption method, alkali neutralization absorption method, and the selective catalytic oxidation absorption method (SCO). At present, the most commonly used SCR method to remove NO_x_ from the flue gas is to reduce NO to N_2_ using NH_3_ as the reducing agent, but the requirement for catalyst is very high. The catalyst in SCR requires a high temperature to produce a good catalytic effect. Therefore, in order to meet the temperature demand of the catalyst, the SCR reactor must be placed in a high temperature area. This makes the catalyst more susceptible to erosion and poisoning from the corrosive substances in high-concentration soot, thereby reducing the service life of the catalyst, and increasing the operating cost of the industry. Additionally, gas treated by the SCR method contains an excessive amount of NH_3_. In view of these defects in the SCR method, this paper mainly studies the selective catalytic oxidation (SCO) method for removing NO. This method can be carried out in a low temperature environment, which greatly reduces energy consumption and costs [3,4].

Selective Catalytic Oxidation (SCO) can, not only simultaneously desulfurize and denitrify, but also produce cost-effective by-products. It has great competitiveness in industrial applications. In the SCO denitration process, NO in the flue gas is oxidized to NO_2_ by the oxygen in the flue gas, under the action of the catalyst, so that the degree of oxidation (NO_2_/NO_x_) reaches 50–60% (at this time the absorption efficiency is the highest). Then, the wet desulfurization absorption process is used for spraying to simultaneously achieve wet desulfurization and denitration. The SCO method can achieve simultaneous desulfurization and denitration with a wet spray absorption process, and this method utilizes the auto-oxidation of NO_x_ and SO_x_ to generate valuable by-products, such as ammonium sulfate. This process has lower investment and operating costs, and when combined with downstream wet spray absorption, the treatment efficiency can reach over 99% [5].

NO can be oxidized to NO_2_ by oxygen in the air without a catalyst, but the NO concentration in coal-fired flue gas is low. As the concentration of NO increases, the rate of NO oxidation to NO_2_ also increases [6,7,8]. Therefore, it is very difficult to oxidize NO to NO_2_ by spontaneous reaction alone. The SCO absorption method has a catalytic oxidation process, and this process greatly increases the denitration rate. If the catalyst used in the SCO process has good performance, the conversion ratio of NO will be high [9]. As a result, the subsequent wet spray absorption process will be more effective, and the NO_x_ removal rate can also reach higher standards. Therefore, for the SCO denitration method, it is very important to select the appropriate catalyst [10,11].

The most widely used carriers for denitration catalysts are molecular sieves and activated carbon. These two carriers have a highly efficient catalytic effect after loading with active metal components, but they have high temperature requirements. In this paper, the dried sludge produced in the sewage treatment process of soybean oil plants (referred to as oil sludge for short) is used as a catalyst carrier. The C/N ratio of the oil sludge is 9.64, and the C/N ratio of urban sludge is 10–20. According to previous research, the smaller the carbon–nitrogen ratio, the better the denitration efficiency of the catalyst. According to Inductive Coupled Plasma Emission Spectrometer (ICP) heavy metal detection, the oil sludge contains more than 50 kinds of metal components, and these metal oxides can very well improve the catalytic activity of the catalyst [12,13,14,15].

The dried sludge contains a large amount of organic matter, and its surface contains a large number of pore-like structures. However, if it is prepared as a denitration catalyst carrier, the organic matter is easily generated in a heated environment, thereby affecting the removal of NO by the catalyst. Therefore, before preparation of the catalyst, the dried oil sludge is alkali activated. This process will corrode a large amount of the organic matter in the oil sludge, and etch more pore-like structures on the surface. Moreover, after being adsorbed on the surface of the alkali activated dry sludge, NO reacts with O_2_ to form NO_2_ [16]. The generated NO_2_ is easily adsorbed by a basic functional group. This will result in a good desorption of NO_2_ on the surface of the sludge, eventually releasing the active sites used to adsorb NO. This promotes the continuous adsorption of NO on the surface of the sludge, so that the reaction continues, and the denitration rate is significantly improved [17,18,19].

Numerous studies have shown that catalysts loaded with metal oxides have better stability in the reaction. Moreover, these catalysts have high catalytic activity. The traditional catalysts are mostly made of TiO_2_ as a catalyst carrier, and loaded with active components such as vanadium and tungsten. However, the adaptation temperature of this catalyst is around 400 °C, and it cannot meet the requirement of low temperature denitration. At 150 °C, only Mn showed significant activity. The high catalytic activity of manganese oxide is related to the valence electron structure of manganese. The electronic structure of manganese is 3d^5^4s^2^, and there are 7 valence electrons. Manganese has more variable valence states than other transition metals. Moreover, it is easy to switch between oxidation states under low temperature conditions. This will lead to better low temperature catalytic activity of manganese in the SCO reaction [20,21,22,23].

The conventional preparation method of the catalyst is the impregnation method. This method must adopt high temperature roasting, where the roasting temperature is generally above 400 °C. The dried sludge is often ignited during this high-temperature roasting process, so the dried sludge must be roasted in the absence of oxygen. In addition, the roasting process is often difficult to control. This makes the prepared catalyst particularly easy to agglomerate, and makes the distribution of active components in the catalyst non-uniform. Based on the above problems, a low-temperature plasma roasting method was used to prepare the sludge catalyst. A large number of studies have shown that after plasma treatment, the surface of the catalyst becomes rough due to etching. In addition, the distribution of the surface active components of the catalyst have an important influence on the catalytic activity. Many researchers have found that plasma affects certain specific structures of the catalyst [24,25]. For example, plasma roasting increases the number of active sites of the catalyst. In addition, plasma roasting enhances the strong interaction between the supported catalyst metal and the catalyst support, forming a special metal-support interface. This will increase the electron transfer efficiency between the semiconductor and the metal, and will give the catalyst a very high catalytic activity. Finally, plasma roasting produces new functional groups on the catalyst surface [26].

In this paper, low temperature plasma roasting is used instead of muffle furnace roasting. The energy of the particles in the low-temperature plasma is generally several to several tens of electron volts, and after the reaction of the material, the chemical bonds of the molecules on the surface of the material can be broken to form a new bond. This will increase the chemical reactivity of the particles, and combine them with free radicals, such as oxygen and nitrogen, in the discharge space. This process creates oxygen and nitrogen containing functional groups on the surface of the material. Studies have shown that the following physicochemical changes may occur after the plasma acts on the surface of the material. Firstly, the active particles strike the surface of the material. This process causes the chemical bonds between the surface molecules to open, generating macromolecular radicals, which cause activity on the surface of the material. Secondly, the plasma etches the surface of the material. The high-energy particles strike the surface of the material to cause physical etching, and the active particles chemically react with the surface of the material to generate chemical erosion.

Therefore, by combining the advantages of the above plasma treatment methods, the physical and chemical properties of the catalyst are greatly optimized, after being treated in low-temperature plasma. Carriers carrying active ingredients, such as metals, are directly placed in a plasma reactor for reduction or oxidation. This treatment method can not only maintain the catalyst skeleton, remove organic impurities such as template, and prevent the sintering of metal clusters from becoming large, but also has a relatively short processing time compared to conventional roasting. Many experiments have shown that this plasma roasting can replace conventional high-temperature roasting [27,28,29].

## 2. Materials and Methods

### 2.1. Materials

The experimental raw material for this study was dried oil sludge from the Xi’an Bangqi Oil Technology Company Wastewater Treatment Station (Xi’an, China), and the results of elemental analysis, and ICP analysis, of the raw materials are shown in Table 1 and Table 2. 

### 2.2. Catalyst Pretreatment

The surface of strong alkali-activated sludge still contains a large amount of organic matter. When plasma is used to roast the catalyst, the organic matter on the surface of the sludge is consumed first. This makes it impossible for the manganese salt loaded on the surface of the sludge catalyst, to be completely oxidized to manganese oxide. Therefore, before the manganese salt is loaded, the raw sludge is first roasted in a muffle furnace so as to consume most of the organic matter on the surface of the sludge. Studies have shown that the sludge catalyst has better denitration performance after being roasted at 450 °C for 1 h in a muffle furnace. Therefore, before the sludge catalyst is loaded with manganese salt, the muffle furnace is used to roast the raw sludge at 450 °C for 1 h, to consume most of the organic matter on the sludge surface.

### 2.3. Catalyst Preparation

The sludge is air-dried first and then crushed into fine particles. A certain quantity of sludge particles (particle size of 2 mm) were selected for use, placed in a muffle furnace (Shanghai Shiyan Electric Furnace Factory, Shanghai, China), and roasted at a temperature of 450 °C for 1 h. Then different concentrations of LiOH activator (5%, 10%, 15%, 20%) were used, and different activation times (2 h, 3 h, 4 h, 5 h) chosen, to prepare modified sludge catalyst. The optimum activation conditions are selected by the catalyst evaluation device. 

The sludge obtained from roasting at 450 °C for 1 h in the muffle furnace was selected. After that, the roasted sludge is subjected to LiOH activation under the above selected optimum activation conditions. Subsequently, the manganese salt is loaded on the catalyst using the equal volume impregnation method. Finally, the catalyst loaded with manganese salt was placed in plasma (Suman Plasma Co., Ltd., Nanjing, China) and roasted. The roasting power was 90 watts, and the roasting time was 9 min. A molded sludge denitration catalyst that loads 2% manganese oxide was prepared (The AP catalyst was used to replace the catalyst prepared by plasma roasting after LiOH activation).

The sludge catalyst obtained from roasting at 450 °C for 1 h in the muffle furnace is selected. After that, manganese salt was loaded onto the catalyst using the equal volume impregnation method. Subsequently, the catalyst loaded with manganese salt was placed in plasma and roasted. The roasting power was 90 watts, and the roasting time was 9 min. Finally, the sludge catalyst is subjected to LiOH activation under the above selected optimum activation conditions. A molded sludge denitration catalyst that loads 2% manganese oxide was prepared (The PA catalyst was used to replace the catalyst prepared by LIOH activation after plasma roasting). 

### 2.4. Catalyst Evaluation Device

**Figure 1:**
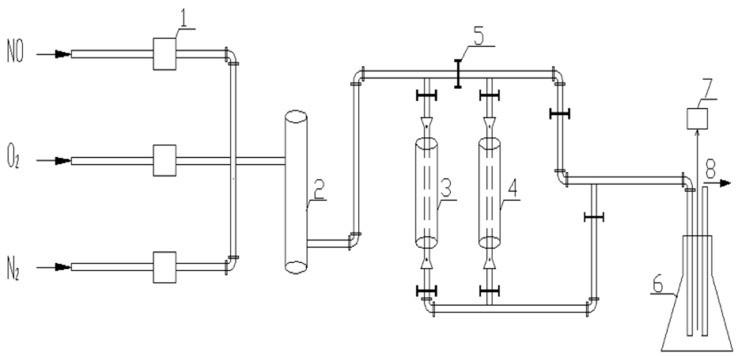
Catalyst activity evaluation experiment process. 1—Flow meter; 2—Mixing tank; 3—Reaction tower A; 4—Reaction tower B; 5—Valve control; 6—gas cylinder; 7—Flue gas analyzer; 8—Exhaust gases.

Figure 1 is a process diagram of a catalyst activity evaluation experiment.

Catalyst activity evaluation means that the catalysts obtained by different preparation methods are evaluated through a flue gas simulation device. First, the total gas flow rate into the device was set to 1000 mL/min, the NO flow rate was set to 20 mL/min, the O_2_ flow rate was set to 60 mL/min, and the N_2_ flow rate was set to 920 mL/min. The three gases are then passed into a mixing tank for mixing. Subsequently, the mixed gas is passed to a reaction tower equipped with catalyst to carry out the denitration reaction. Finally, the catalyzed gas is passed into the gas cylinder. The gas is detected using the flue gas analyzer, and the remaining gas is discharged. In this experiment, the NO concentration was measured using the Flue gas analyzer (Testo340, Mingle Instrument, Guangzhou, China). The Flue gas analyzer is a custom instrument with NO and O_2_ modules. It can detect NO and O_2_. The amount of catalyst in the reaction is 3 g, the catalyst can build up a 2 cm high reaction layer, and it can make a good contact with simulated flue gas and catalyst. The Set temperature of the reaction tower is 150 °C. 

The NO conversion ratio is calculated as follows: The initial NO concentration of the flue gas (concentration A) passed into the reaction system is obtained using the flow meter, and then the NO concentration of the flue gas (concentration B) after the catalytic reaction, is obtained using the flue gas analyzer. A-B/A is the NO conversion ratio.

**Figure 2:**
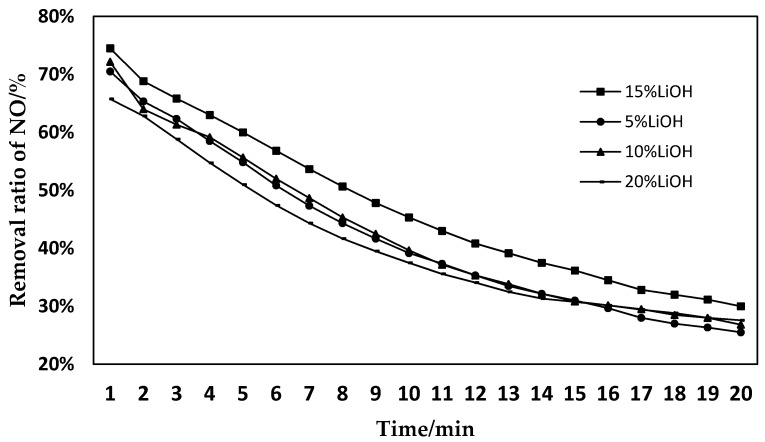
Effects of sludge with different concentrations of LiOH activation on the removal of NO.

**Figure 3:**
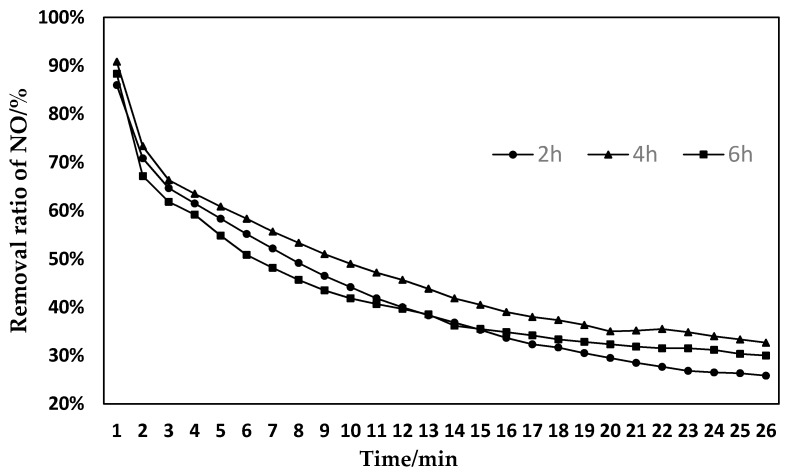
Effects of sludge with different activation times of LiOH on the removal of NO.

The sludge was activated for 2 h using 5% LiOH, 10% LiOH, 15% LiOH, and 20% LiOH, and then the denitration experiment was performed on the modified sludge. The conversion ratio of NO is shown in Figure 2. The sludge was activated for 2 h, 4 h, and 6 h, using a concentration of 15% LiOH, and then the denitration experiment was performed on the modified sludge. The conversion ratio of NO is shown in Figure 3.

## 3. Results and Discussion

### 3.1. Effects of Different Activation Concentrations on the Denitration Performance of SCO

From Figure 2, it can be seen that the sludge has the best NO removal effect after being activated with 15% LiOH. If the concentration of LiOH is too high or too low, the removal effect of activated sludge on NO will be reduced. Within 2–17 min of the reaction, the NO removal rate of sludge activated with 15% LiOH was significantly higher than that of sludge activated with other concentrations. The reason is that when the LiOH concentration is too low, no effective active sites can be formed on the surface of the sludge, and therefore the NO oxidation reaction is not significant. With the increase of alkali concentration, the active sites for the oxidation reaction gradually increase, which improves the reaction efficiency of NO and O_2_, and significantly enhances the denitration effect. However, as the concentration of LiOH continues to increase, the activation of sludge by LiOH reaches saturation. Too much alkali will destroy the existing active sites, resulting in obstruction of the oxidation reaction, and reduced denitration efficiency. Therefore, the optimal LiOH activation concentration was 15% [30].

### 3.2. Effect of Different Activation Times on SCO Denitration Performance

As can be seen from Figure 3, the NO removal effect of sludge activated by LiOH for 4 h is the best, and the NO removal effect of sludge activated for 6 h is the worst. Within 10–20 min of the reaction, sludge activated after 4 h had a significantly higher NO removal rate and continuity, than sludge activated after 2 h and 6 h. The reason is that when the alkali activation time is 2 h, the activation time is too short, and LiOH and the sludge cannot fully react. The amount of effective pore structure formed by the sludge is less, so the desired activation effect cannot be achieved, and sufficient active sites cannot be formed. As the activation time increases, the activation efficiency becomes higher and higher, and the degree of activation gradually deepens. As a result, more and more effective pores have been formed, so the denitration effect has gradually increased. However, as the activation time becomes longer, LiOH will destroy the previously formed micro-porous structure. The pores collapse, destroying the original effective pore location. Therefore, the effect of denitration decreased significantly. In summary, the appropriate activation time is particularly advantageous for the activation effect. If the time is too long or too short, proper active sites cannot be formed. Therefore, the optimal activation time for screening LiOH was 4 h [31].

### 3.3. Effect of Different Preparation Sequences on Catalytic Activity of Catalysts

**Figure 4:**
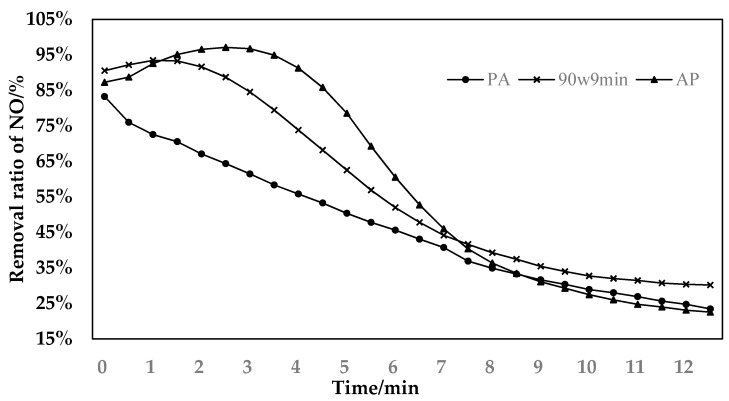
Comparison of catalytic activity of catalysts prepared separately with different activation sequences and plasma.

As can be seen from Figure 4, the denitration catalyst prepared by plasma roasting after alkaline activation is effective in removing NO. It is obviously better than the catalyst prepared by plasma roasting before alkaline activation. It is also significantly better than the denitration catalyst prepared only by plasma roasting. The catalytic activity of the catalyst prepared by plasma roasting before activation reaches over 85% in the reaction, and is far better than the other two prepared catalysts. The reason is, after the sludge was roasted in a muffle furnace at a temperature of 450 °C for 1 h, the organic matter on its original sludge surface was almost consumed. Subsequently, the sludge was activated with LiOH at a mass concentration of 15% for 4 h. This process can form a large amount of –OH basic functional groups on the surface of the sludge. According to the previous experimental results, with the increase of the number of –OH basic functional groups on the surface of the sludge catalyst, the activity of the sludge denitration catalyst improves in the catalytic oxidation of NO. After the PA catalyst is calcined by the plasma, manganese oxides are formed on the surface of the sludge catalyst. However, after being activated by LiOH, it is washed off with water. This results in a reduction of the manganese oxide on the surface of the final sludge catalyst, and a reduction in the active sites. As a result, the catalytic activity of the catalyst is low [32].

## 4. Characterization

### 4.1. Infrared Spectrum Analysis

**Figure 5:**
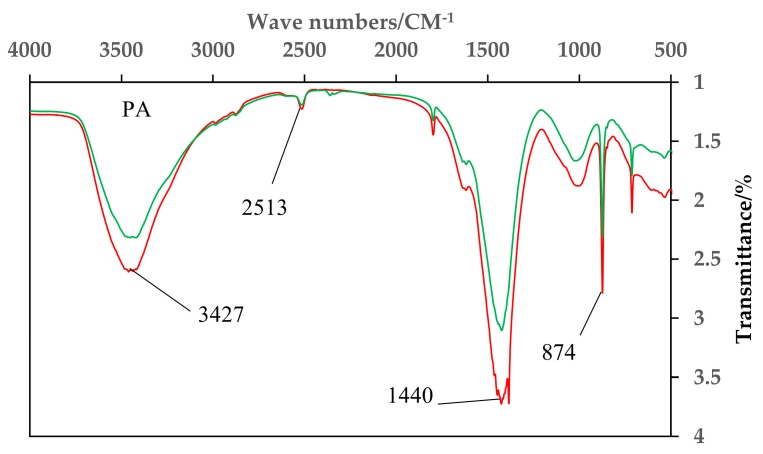
Infrared spectrum of catalysts prepared with different activation sequences.

From Figure 5, there is a stretching vibration band of –NO at 874cm^–1^, and a stretching vibration band of –NO_2_ at 1440cm^–1^. Both of these groups are from the organic matter in the sludge. The catalyst prepared by plasma roasting before LIOH activation (referred to as PA catalyst) has a weaker stretching vibration of the –OH functional group. Based on this, it can be speculated that this is the oxidation mechanism of SCO. The denitration efficiency of the PA catalyst is low. The reason is that the concentration of basic functional groups on the surface of the catalyst is low, so NO_2_ cannot be completely adsorbed by the basic functional group. When all the NO adsorption sites are covered by the NO_2_ formed in the late stage of the reaction, the denitration effect is significantly reduced. The AP catalyst however, has high denitration efficiency. This is due to the high concentration of surface-OH basic functional groups of the sludge catalyst. Therefore, NO_2_ is easily adsorbed by a basic functional group, thereby releasing a large amount of NO adsorption sites. This process promotes the continuous adsorption of NO on the sludge surface, and the denitration rate is significantly improved [33].

### 4.2. XPS Analysis

**Figure 6:**
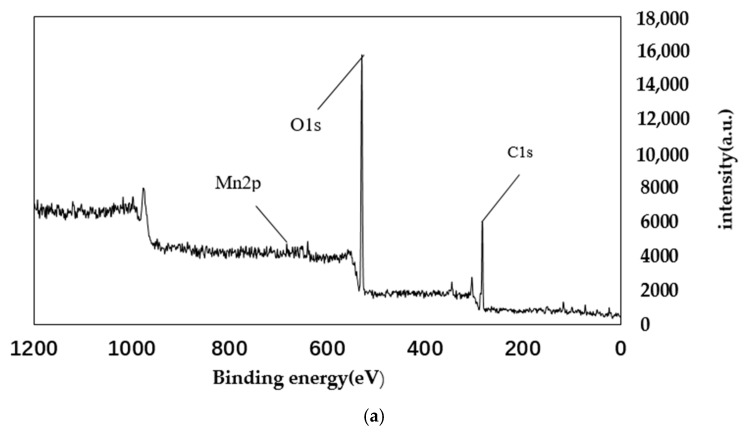

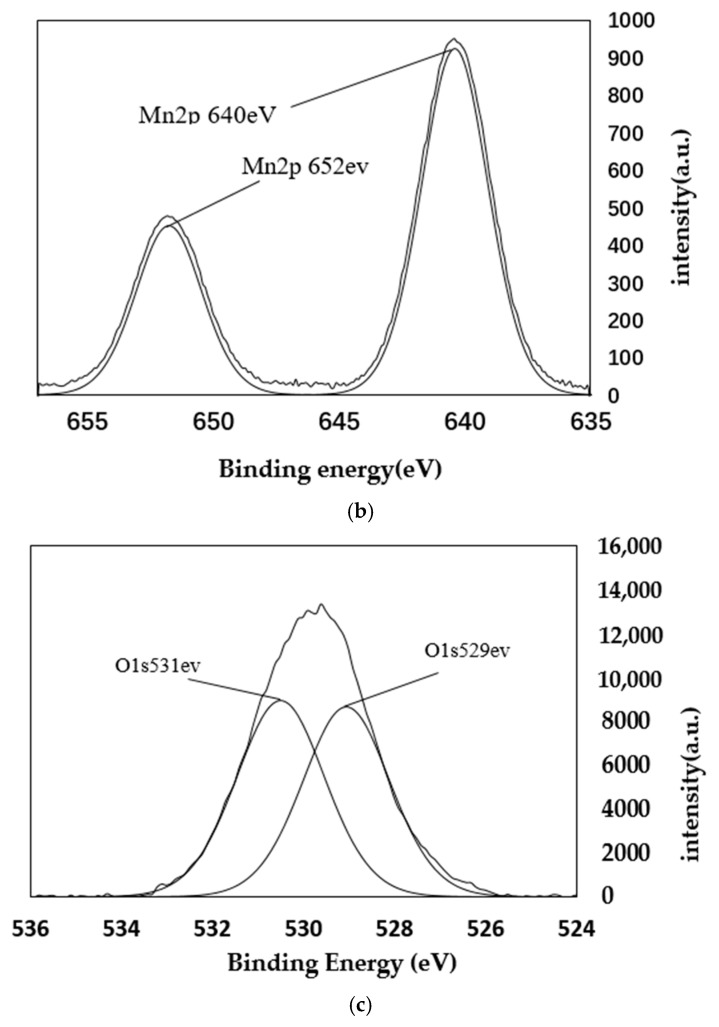
XPS spectra of AP catalyst before reaction. (**a**) Total element XPS spectrum; (**b**) XPS spectra of Mn; (**c**)XPS spectra of O.

**Figure 7:**
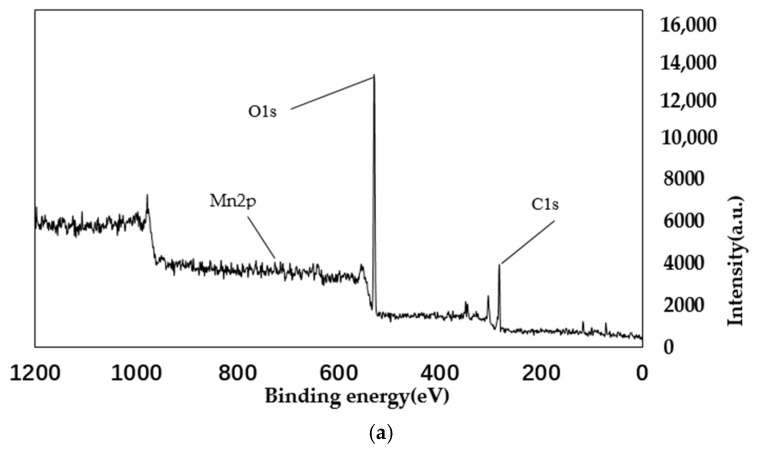

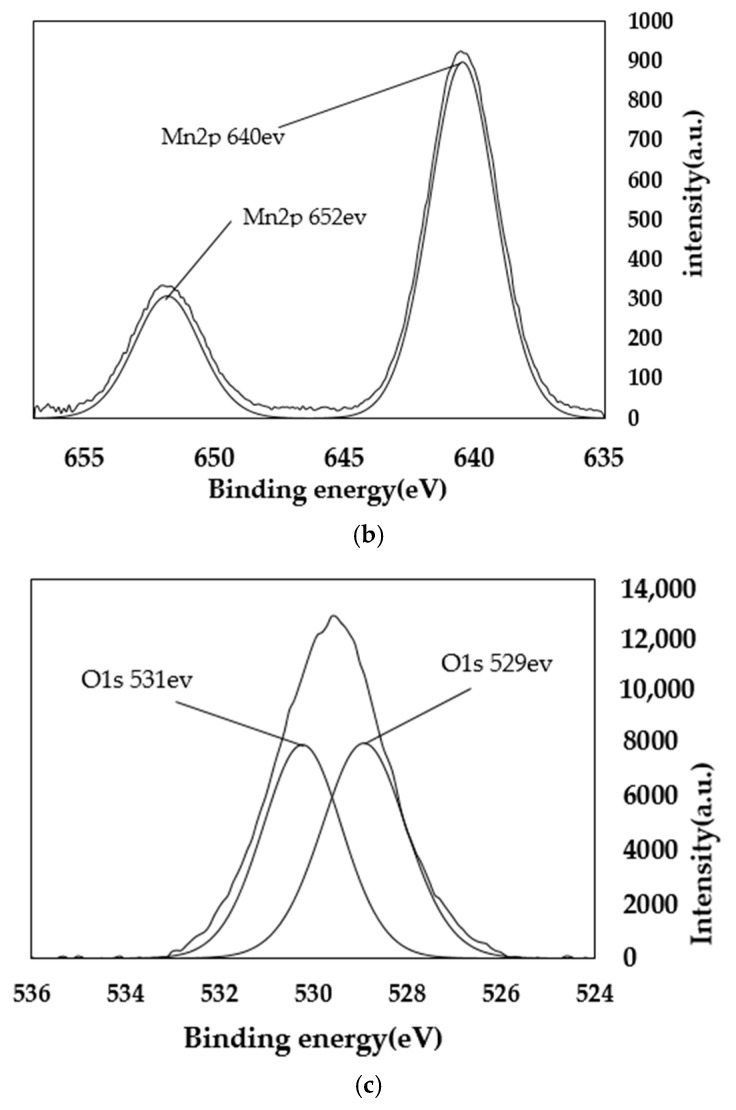
XPS spectra of AP catalyst after reaction. (**a**) Total element XPS spectrum; (**b**)XPS spectra of Mn; (**c**)XPS spectra of O.

From the main spectra of Figure 6 and Figure 7, it can be seen that the main elements contained in the AP catalyst are oxygen, carbon, and manganese. The manganese is loaded by equal volume impregnation. As can be seen from the Mn spectrum, there are two peaks of manganese metal on the surface of the catalyst. The two peaks are Mn2p3/2 and Mn2p1/2. This shows that there are two manganese oxides with different valences in the catalyst. Among them, the Mn2p3/2 peak, with a binding energy of 640 eV, represents MnO_2_; and the Mn2p1/2 peak, with a binding energy of 652 eV, represents Mn_2_O_3_. As can be seen from the figure, before and after the reaction, the main Mn oxides formed in AP catalyst are MnO_2_ and Mn_2_O_3_. Comparing Figure 6 and Figure 7, it can be seen that as the reaction proceeds, the amount of Mn^4+^ increases, while the amount of Mn^3+^ decreases, eventually leading to a decrease in denitration efficiency. It can be concluded that Mn_2_O_3_ plays a major role in the reaction. From the spectrum in Figure 6c of the O element, it can be seen that the lattice oxygen is at 529 eV, and the chemisorbed oxygen is at 531 eV. It can be seen that lattice oxygen accounts for a large proportion. It can also be seen that chemisorbed oxygen accounts for a large proportion. Comparing Figure 6 and Figure 7, it can be seen that the lattice oxygen and the chemisorbed oxygen are greatly reduced after the reaction. However, the reduction ratio of chemisorbed oxygen is less than the reduction ratio of lattice oxygen. This shows that it is mainly lattice oxygen that plays a role during the reaction [34,35].

**Figure 8:**
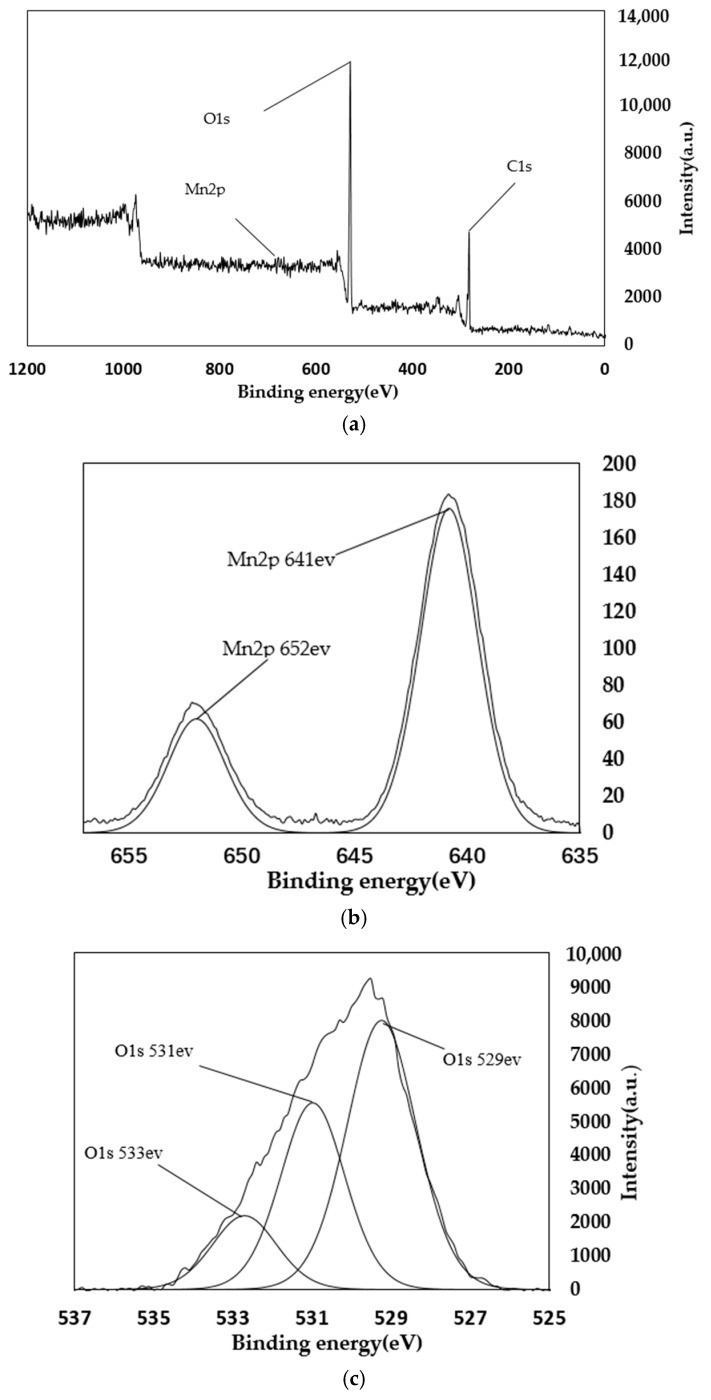
XPS spectra of PA catalyst before reaction. (**a**) Total element XPS spectrum; (**b**)XPS spectra of Mn; (**c**)XPS spectra of O.

**Figure 9:**
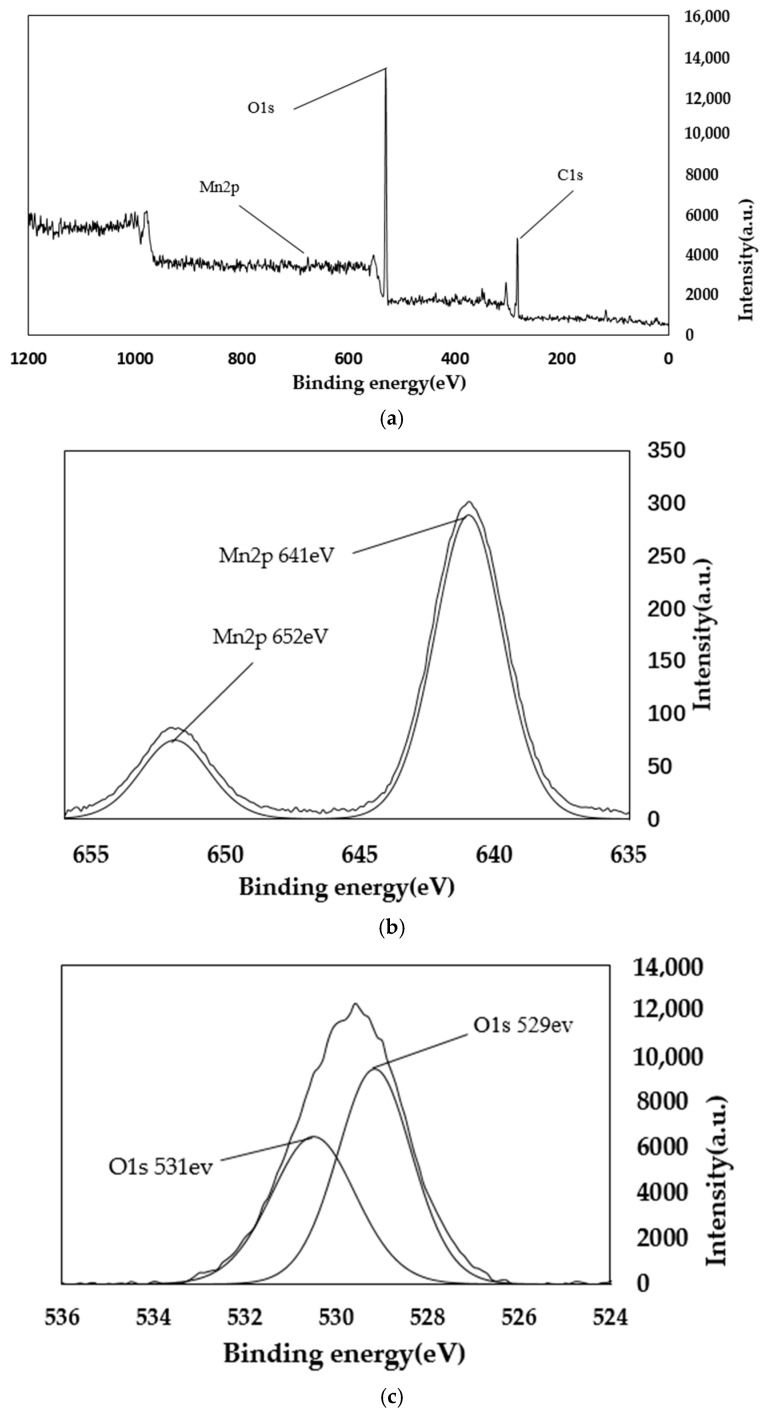
XPS spectra of PA catalyst after reaction. (**a**) Total element XPS spectrum; (**b**)XPS spectra of Mn; (**c**)XPS spectra of O.

From the main spectra of Figure 8 and Figure 9, it can be seen that the main elements contained in the PA catalyst are oxygen, carbon, and manganese. The manganese is loaded by equal volume impregnation. As can be seen from the Mn spectrum, there are two peaks of manganese metal on the surface of the catalyst. The two peaks are Mn2p3/2 and Mn2p1/2. This shows that there are two manganese oxides with different valences in the catalyst. Among them, the Mn2p3/2 peak, with a binding energy of 641 eV, represents MnO_2_; and the Mn2p1/2 peak, with a binding energy of 652 eV, represents Mn_2_O_3_. As can be seen from the figure, before and after the reaction, the main Mn oxides formed in AP catalyst are MnO_2_ and Mn_2_O_3_. Comparing Figure 8 and Figure 9, it can be seen that as the reaction proceeds, the amount of Mn^4+^ increases, while the amount of Mn^3+^ decreases, eventually leading to a decrease in denitration efficiency. It can be concluded that Mn_2_O_3_ plays a major role in the reaction. From the spectrum in Figure 8c of the O element, it can be seen that the lattice oxygen is at 529 eV, and the chemisorbed oxygen is at 531 eV and 533 eV. It can be seen that lattice oxygen accounts for a large proportion. It can also be seen that chemisorbed oxygen accounts for a large proportion. Comparing Figure 8 and Figure 9, it can be seen that the lattice oxygen and the chemisorbed oxygen are greatly reduced after the reaction. However, the reduction ratio of chemisorbed oxygen is less than the reduction ratio of lattice oxygen. This shows that it is mainly lattice oxygen that plays a role during the reaction [36,37]. 

### 4.3. XRD Analysis

**Figure 10:**
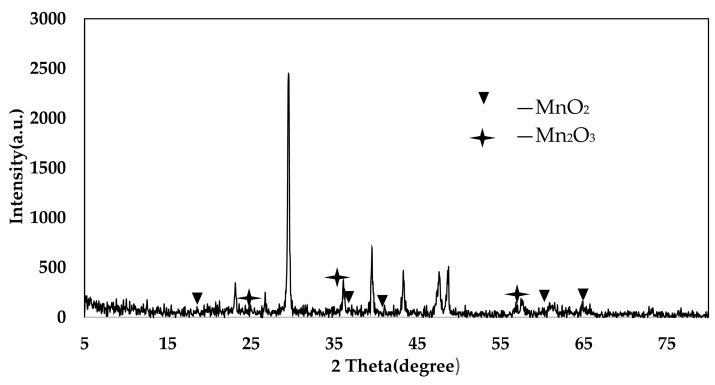
XRD spectra of AP catalyst.

From the XRD pattern of the AP catalyst in Figure 10, it can be seen that the characteristic peaks of the MnO_2_ crystal form appear at the θ angles of 20.99°, 37.83°, 41.57°, 41.6°, 60.16°, and 65.93°. The characteristic peaks of the Mn_2_O_3_ crystal form appear at positions of 25.13°, 35.24°, and 53.55°. It can be concluded that the Mn oxides on the surface of the AP sludge denitration catalyst are mainly MnO_2_ and Mn_2_O_3_. These indicate that the prepared catalyst surface has a good Mn oxide crystal form. This enables the sludge catalyst to have good catalytic activity for the selective catalytic oxidation of NO [38,39].

**Figure 11:**
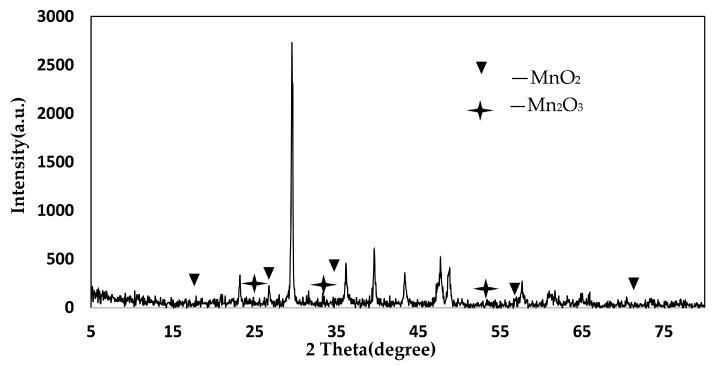
XRD spectra of PA catalyst.

From the PA catalyst XRD pattern in Figure 11, it can be seen that the characteristic peaks of the MnO_2_ crystal form appear at the θ angles of 17.78°, 28.83°, 37.73°, 57°, and 70.88°. The characteristic peaks of the Mn_2_O_3_ crystal form appear at the θ angles of 25.01°, 33.81°, and 50.85°. It can be concluded that the Mn oxides on the PA catalyst surface are mainly MnO_2_ and Mn_2_O_3_. These indicate that the prepared catalyst surface has a good Mn oxide crystal form. This enables the sludge catalyst to have good catalytic activity for the selective catalytic oxidation of NO [40].

### 4.4. XRD Analysis

**Figure 12:**
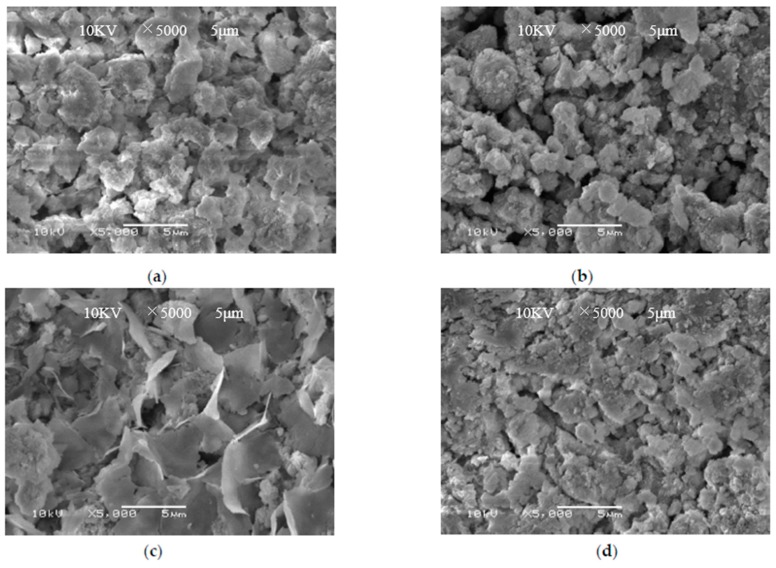
Scanning electron microscope image of catalyst. (**a**) AP catalyst before reaction; (**b**) After AP catalyst reaction; (**c**) PA catalyst before reaction; (**d**) After PA catalyst reaction.

Scanning electron microscopy analysis of the AP catalyst before and after the reaction was performed, and scanning electron microscopy analysis of the PA catalyst before and after the reaction was performed. The magnification was 5000 times. The results are shown in Figure 12.

From Figure 12a–d, it can be seen that before and after the AP catalyst reaction, there are obvious Mn oxide crystal grains on the surface. However, no significant particulate matter was found on the surface of the PA catalyst before and after the reaction. The number of active sides on the surface of Mn oxides in the PA catalyst is less, resulting in a low NO removal rate for the PA catalyst.

### 4.5. Elemental Analysis

According to Table 3 and Table 4, it can be seen that the content of MN in the catalyst increases after the load.

## 5. Conclusions

In this paper, oil sludge was used as catalyst carrier, and LiOH was used to modify the carrier. The optimal concentration of modified LiOH catalyst, and the optimal modification time were selected. The effect of modified sludge conditions on the denitration rate was investigated. Subsequently, two catalysts with different activation sequences were prepared. The effect of different activation sequences on the catalytic performance of the sludge denitration catalyst was investigated. We can draw the following conclusions:

1. Catalysts activated with LiOH at a concentration of 15% have better denitration effects.
2. Catalysts activated with LIOH for 4 h have better denitration effects.
3. The sludge denitration catalyst (AP catalyst) prepared by plasma after being activated first, has a better denitration effect.

## Figures and Tables

**Table 1:** Elemental analysis of dried sludge.

Sample Mass	Nitrogen Content/%	Carbon Content/%	Hydrogen Content/%	C/N
3.931	1.568	15.12	2.549	9.64

**Table 2:** ICP analysis of dried sludge.

>5 ppm	1~5 ppm	0.1~1 ppm	<0.1 ppm
Al Ca Mg Fe Na P S Si	B K Sr Ti Zn.	Ag Ba Mn Ni Pd	As Be Cd Ce Cr Cu and other 30 kinds

**Table 3:** Elemental analysis of PA catalyst.

Sample Mass	Nitrogen Content/%	Carbon Content/%	Hydrogen Content/%	C/N	Manganese Content/%
3.769	1.543	15.097	2.556	9.784	1.264

**Table 4:** Elemental analysis of AP catalyst.

Sample Mass	Nitrogen Content/%	Carbon Content/%	Hydrogen Content/%	C/N	Manganese Content/%
3.761	1.559	15.063	2.511	9.661	1.271

## References

[1] Wang S.X., Guo R.T., Pan W.G., Li M.Y., Sun P., Liu S.M., Liu S.W., Sun X., Liu J. (2017). The deactivation mechanism of Pb on the Ce/TiO_2_ catalyst for the selective catalytic reduction of NOx with NH_3_: TPD and DRIFT studies. Phys. Chem. Chem. Phys..

[2] Zhou Y., Li C.T., Fan C.Z., Fu M.F., Tao L., Yu M.G., Zhang M.Y. (2016). Wet removal of sulfur dioxide and nitrogen oxides from simulated flue gas by Ca(ClO)_2_ solution. Environ. Prog. Sustain..

[3] Li P.X., Zhang R.D., Liu N., Royer S. (2017). Efficiency of Cu and Pd substitutionin Fe-based perovskites to promote N_2_ formation during NH_3_ selective catalytic oxidation(NH_3_-SCO). Appl. Catal. B-Environ..

[4] Guo R.T., Wang S.X., Pan W.G., Li M.Y., Sun P., Liu S.M., Sun X., Liu S.W., Liu J. (2017). Different poisoning effects of K and Mg on the Mn/TiO_2_ catalyst for selective catalytic reduction of NO_X_ with NH_3_: A mechanistic study. J. Phys. Chem. C.

[5] Zhao N., Li S., Wang J. (2015). Synthesis and application of different phthalocyanine molecular sieve catalyst for oxidative desulfurization. J. Solid State Chem..

[6] Hu Y., Griffiths K. (2017). Selective catalytic reduction of NO in the presence of SO_2_ and O_2_: The poisoning effect of SO_x_ under oxygen rich and lean conditions. Surf. Sci..

[7] Zeng X., Liu J., Zhao J. (2018). Catalytic wet oxidation of pharmaceutical sludge by molecular sieve loaded with Cu/Ce. Catalysts.

[8] Zhang R., Wang C., Li K., Sun X., Ning P., Tang L.H., Liu Y. (2017). Influence of Ca doping and calcination temperature on selective catalytic oxidation of NO over Mn-Ca-O_x_-(CO_3_)_y_ catalyst. New J. Chem..

[9] Ramis G., Yi L., Busca G. (1996). Ammonia activation over catalysts for the selective catalytic reduction of NO_x_, and the selective catalytic oxidation of NH_3_. An FT-IR study. Catal. Today.

[10] Zhang X.Y., Wang H., Wang Z., Qu Z.P. (2018). Adsorption and surface reaction pathway of NH_3_, selective catalytic oxidation over different Cu-Ce-Zr catalysts. Appl. Surf. Sci..

[11] Wang F., Ma J.Z., He G.Z., Chen M., Zhang C.B., He H. (2018). Nanosize effect of Al_2_O_3_ in Ag/Al_2_O_3_ catalyst for the selective catalytic oxidation of ammonia. ACS Catal..

[12] Zhou G.Q., Fang F., Chen Z.W., He Y.F., Sun H.L., Shi H.X. (2015). Facile synthesis of paper mill sludgederived heterogeneous catalyst for the Fenton-like degradation of methylene blue. Catal. Commun..

[13] Lu S.Y., Liu Y.Z., Li F., Sun Z.G., Zhang L.Q. (2018). Characterization of ferromagnetic sludge-based activated carbon and its application in catalytic ozonation of p-chlorobenzoic acid. Environ. Sci. Pollut. Res..

[14] Guo S., Yuan N., Zhang G., Yu J.C. (2017). Graphene modified iron sludge derived from homogeneous Fenton process as an efficient heterogeneous Fenton catalyst for degradation of organic pollutants. Microporous Mesoporous Mater..

[15] Xie Q., Peng P., Liu S., Min M., Cheng Y., Wan Y., Li Y., Liu X., Liu Y., Chen P. (2014). Fast microwave-assisted catalytic pyrolysis of sewage sludge for bio-oil production. Bioresour. Technol..

[16] Hu M., Lan G., Chen Z., Ma C., Zhou Y., Chen J., Ma S., Laghari M., Xiao B., Zhang B. (2016). Syngas production by catalytic in-situ steam co-gasification of wet sewage sludge and pine sawdust. Energy Convers. Manag..

[17] Liu J.J., Diao Z.H., Liu C.M., Jiang D., Kong L.J., Xu X.R. (2018). Synergistic reduction of copper (II) and oxidation of norfloxacin over a novel sewage sludge-derived char-based catalyst: Performance, fate and mechanism. J. Clean. Prod..

[18] Su C., Li W., Chen M., Huang Z., Wu L. (2016). Effect of iron-manganese-sepiolite as heterogeneous Fenton-like catalyst on the performance and microbial community of anaerobic granular sludge treatment system. Bioresour. Technol..

[19] Faheem M., Jiang X., Wang L., Shen J. (2018). Synthesis of Cu_2_O-CuFe_2_O_4_ microparticles from Fenton sludge and its application in the Fenton process: The key role of Cu_2_O in the catalytic degradation of phenol. RSC Adv.

[20] Chen Q.L., Guo R.T., Wang Q.S., Pan W.G., Wang W.H., Yang N.Z., Lu C.Z., Wang S.X. (2016). The catalytic performance of Mn/TiWO_x_, catalyst for selective catalytic reduction of NO_x_, with NH_3_. Fuel.

[21] Wu Z., Jin R., Liu Y., Wang H. (2008). Ceria modified MnO_x_/TiO_2_ as a superior catalyst for NO reduction with NH_3_ at low-temperature. Catal. Commun..

[22] Qi G., Yang R.T., Chang R. (2004). MnOx–CeO_2_, mixed oxides prepared by co-precipitation for selective catalytic reduction of NO with NH_3_, at low temperatures. Appl. Catal. B-Environ..

[23] Li S., Huang B., Yu C. (2017). A CeO_2_-MnOx, core-shell catalyst for low-temperature NH_3_-SCR of NO. Catal. Commun..

[24] Zhang J., Zhang J., Xu Y., Su H., Li X., Zhou J., Qian G., Li L., Xu Z. (2014). Efficient selective catalytic reduction of NO by novel carbon-doped metal catalysts made from electroplating sludge. Environ. Sci. Technol..

[25] Jogi I., Erme K., Levoll E., Raud J., Stamate E. (2018). Plasma and catalyst for the oxidation of NO_x_. Plasma Sources Sci. Technol..

[26] Li G., Zhu B., Sun Y., Yin S., Zi Z., Fang Q., Ge T., Li J. (2018). Study of the alkali metal poisoning resistance of a Co-modified Mn/Ni foam catalyst in low-temperature flue gas SCR DeNO_x_. J. Mater. Sci..

[27] Lee B.J., Kang H.C., Jo J.O., Mok Y.S. (2017). Consideration of the role of plasma in a plasma-coupled selective catalytic reduction of nitrogen oxides with a hydrocarbon reducing agent. Catalysts.

[28] Jiang N., Shang K.F., Lu N., Li H., Li J., Wu Y. (2016). High-efficiency removal of NO_x_ from flue gas by multitooth wheel-cylinder corona discharge plasma facilitated selective catalytic reduction process. IEEE Trans. Plasma Sci..

[29] Zhang L., Sha X.L., Zhang L. (2016). Synergistic catalytic removal NO_X_ and the mechanism of plasma and hydrocarbon gas. AIP Adv..

[30] Boningari T., Smirniotis P.G. (2016). Impact of nitrogen oxides on the environment and human health: Mn-based materials for the NO_x_ abatement. Curr. Opin. Chem. Eng..

[31] Cao F., Xiang J., Su S., Wang P., Hu S., Sun L. (2015). Ag modified Mn–Ce/γ-Al_2_O_3_, catalyst for selective catalytic reduction of NO with NH_3_, at low-temperature. Fuel Process. Technol..

[32] O’Brien C.J., Droege D.G., Jiu A.Y., Gandhi S.S., Paras N.A., Olson S.H., Conrad J. (2018). Photoredox cyanomethylation of indoles: Catalyst modification and mechanism. J. Org. Chem..

[33] Wang F., Wang S., Meng Y., Zhang L., Wu Q., Hao J. (2016). Mechanisms and roles of fly ash compositions on the adsorption and oxidation of mercury in flue gas from coal combustion. Fule.

[34] Chen C.H., Njagi E.C., Chen S.Y., Horvath D.T., Xu L., Morey A., Mackin C., Joesten R., Suib S.L. (2015). Structural distortion of molybdenum-doped manganese oxide octahedral molecular sieves for enhanced catalytic performance. Inorg. Chem..

[35] Chandrasekhar S., Kumar C.P., Kumar T.P., Haribabu K., Jagadeesh B., Lakshmi J.K., Mainkar P.S. (2015). ChemInform abstract: Peptidomimetic organocatalysts: Efficient michael addition of ketones onto nitroolefins with very low catalyst loading. RSC Adv..

[36] Jampaiah D., Ippolito S.J., Sabri Y.M., Tardio J., Selvakannan P.R., Nafady A., Reddy N.M., Bhargava S.K. (2016). Ceria-zirconia modified MnO_x_ catalysts for gaseous elemental mercury oxidation and adsorption. Catal. Sci. Technol..

[37] Jampaiah D., Tur K.M., Venkataswamy P., Ippolito S.J., Sabri J.M., Tardio J., Bhargava S.K., Reddy B.M. (2015). Catalytic oxidation and adsorption of elemental mercury over nanostructured CeO_2_–MnO_x_ catalyst. RSC Adv..

[38] Li H., Wu C.Y., Li L., Li Y., Zhao Y., Zhang J. (2013). Kinetic modeling of mercury oxidation by chlorine over CeO_2_–TiO_2_, catalysts. Fuel.

[39] Li H., Wu C.Y., Li Y., Zhang J. (2012). Superior activity of MnO_x_-CeO_2_/TiO_2_, catalyst for catalytic oxidation of elemental mercury at low flue gas temperatures. Appl. Catal. B-Environ..

[40] Li J., Chen J., Yu Y., He C. (2015). Fe–Mn–Ce/ceramic powder composite catalyst for highly volatile elemental mercury removal in simulated coal-fired flue gas. J. Ind. Eng. Chem..

